# Influence of Nanosilica on Mechanical Properties, Sorptivity, and Microstructure of Lightweight Concrete

**DOI:** 10.3390/ma12193078

**Published:** 2019-09-21

**Authors:** Mohamed Abd Elrahman, Sang-Yeop Chung, Pawel Sikora, Teresa Rucinska, Dietmar Stephan

**Affiliations:** 1 Building Materials and Construction Chemistry, Technische Universität Berlin, Gustav-Meyer-Allee 25, 13355 Berlin, Germany; mohamedattia@mans.edu.eg (M.A.E.); stephan@tu-berlin.de (D.S.); 2 Structural Engineering Department, Mansoura University, Elgomhouria St., Mansoura 35516, Egypt; 3 Department of Civil and Environmental Engineering, Sejong University, 209 Neungdong-ro, Gwangjin-gu, Seoul 05006, Republic of Korea; sychung@sejong.ac.kr; 4 Faculty of Civil Engineering and Architecture, West Pomeranian University of Technology, Szczecin, Al. Piastow 50, 70-311 Szczecin, Poland; teresa.rucinska@zut.edu.pl

**Keywords:** lightweight aggregate concrete, nanosilica, cement-based composites, mechanical properties, water absorption, sorptivity, porosity

## Abstract

This study presents the results of an experimental investigation of the effects of nanosilica (NS) on the strength development, transport properties, thermal conductivity, air-void, and pore characteristics of lightweight aggregate concrete (LWAC), with an oven-dry density <1000 kg/m^3^. Four types of concrete mixtures, containing 0 wt.%, 1 wt.%, 2 wt.%, and 4 wt.% of NS were prepared. The development of flexural and compressive strengths was determined for up to 90 days of curing. In addition, transport properties and microstructural properties were determined, with the use of RapidAir, mercury intrusion porosimetry (MIP), and scanning electron microscopy (SEM) techniques. The experimental results showed that NS has remarkable effects on the mechanical and transport properties of LWACs, even in small dosages. A significant improvement in strength and a reduction of transport properties, in specimens with an increased NS content, was observed. However, the positive effects of NS were more pronounced when a higher amount was incorporated into the mixtures (>1 wt.%). NS contributed to compaction of the LWAC matrix and a modification of the air-void system, by increasing the amount of solid content and refining the fine pore structure, which translated to a noticeable improvement in mechanical and transport properties. On the other hand, NS decreased the consistency, while increasing the viscosity of the fresh mixture. An increment of superplasticizer (SP), along with a decrement of stabilizer (ST) dosages, are thus required.

## 1. Introduction

Lightweight concrete (LWC), often utilized in structural and masonry elements, is a versatile construction material with reduced dry density and low thermal conductivity. Moreover, because of the lower thermal conductivity of LWC, compared with normal-weight concrete, LWCs with low densities are widely used as heat and acoustic insulating elements. LWC owes its properties to the presence of natural or artificial aggregates with low specific densities (so-called lightweight aggregate concretes—LWACs), or the induction of air-voids in concrete or mortar (so-called cellular concrete). Depending on its density and the purpose of its use, lightweight concrete can be categorized in three ways: (I) low density concrete with a density lower than 800 kg/m^3^, (II) moderate strength concrete with a density range of 800–1400 kg/m^3^, and (III) structural concrete with a density range of 1400 kg/m^3^–2000 kg/m^3^. However, as a result of its low density and high porosity, attributed to the intrinsic nature of lightweight aggregate or foaming agents, a weak LWC microstructure is produced. Thus, the mechanical performance of LWCs is significantly worse than that of normal-weight concretes [1,2,3,4,5]. However, lightweight concrete is characterized by its low thermal conductivity because of numerous number of pores compared with normal-weight concrete. Both pores in the aggregate and the cement matrix affect thermal properties of lightweight concrete. Owing to its high thermal insulation characteristics, lightweight concrete can be effectively used to save energy in buildings and structures, which requires high thermal resistance.

In recent years, increased interest has thus focused on the development of LWC with satisfactory mechanical and durability performance, through the optimization of packing densities and the incorporation of the proper aggregate type, fillers, and cement additives (mainly ground granulated blast furnace slag (GGBFS), fly ash (FA), and silica fume (SF)) [6]. In addition, particular attention has also been paid to nanosized admixtures, which exhibit higher reactivity than their corresponding materials on the microscale, thus increasing the performance of cement-based composites with a lower required amount of admixture. Noticeable attention has been given to nanosilica (NS), which has a noticeably higher reactivity than the widely used SF [7]. The effects of silica nanoparticles on the properties of cement-based composites have been extensively studied by researchers over the last two decades, but their effects have mostly been studied using normal-weight cement-based composites [8,9,10,11], while the effects of NS on the properties of LWCs have seemingly been overlooked. Compared with other fillers, nanosilica affects the properties of concrete owing to its superior reactivity and very small particle size. The addition of nanosilica can influence the properties of cement-based materials in three ways: the nucleation effect which, accelerates the hydration of cement; the filling effect, which strengthens the microstructure of the material; and the pozzolanic activity, which produces additional C–S–H gel, improving the mechanical properties of concrete [8,9,10].

As far as the authors of this paper are aware, only a limited number of papers have been devoted to a characterization of the effects of NS on the properties of LWACs. Du et al. [12] have evaluated the effects of a low NS admixture dosage (in the amounts of 1% and 2% by weight of cement) on the properties of structural LWACs. The authors reported an improvement in the compressive strength of the LWAC after 1 and 7 days of curing, but after 28 days, the strength remained similar to that of pristine concrete specimens. The incorporation of NS also contributed to a decrement in water accessible porosity, water penetration depth, water sorptivity, as well as to a higher resistance against chloride ion penetration. In the work of Du [13], the effects of 1 wt.%–3 wt.% addition of NS on the properties of LWACs (with densities in the range of 1075–1240 kg/m^3^) showed that an optimal amount of NS (i.e., 2 wt.%) contributed to an improvement in compressive strength development from one day of hydration, as well as to decreased water permeability and chloride penetration. Atmaca et al. [14] have analysed the effects of 3 wt.% of NS cement replacement on the compressive strength, split tensile strength, water sorptivity, and gas permeability of high-strength LWAC. The study showed that NS-incorporated LWACs exhibit higher compressive strength and split-tensile strength for up to 90 days. In addition, a decrement in water sorptivity and gas permeability in NS-incorporated specimens was also observed. The studies undertaken by Zhang et al. [15] have shown that a low NS amount (0.1 wt.%–0.5 wt.%) is beneficial for flexural and compressive strength improvements after 7 and 28 days of curing of LWACs. However, incorporating higher dosages of NS (1 wt.%) leads to neutralisation of its beneficial effects, with NS-modified samples exhibiting comparable strengths to that of the control samples. In contrast to the work mentioned above, Vargas et al. [16] have analyzed the effects of replacing cement with a high dosage of NS (10 wt.%) on the properties of LWACs. The study showed that NS-incorporated specimens exhibit similar 28-day strength to that of pristine concrete. However, because of a refinement of cement paste microstructure by nanosilica, NS-incorporated samples exhibit a lower volume of voids and a decreased water absorption rate.

Previous studies have mainly focused only on the effects of NS on material properties of normal-weight concrete. However, more detailed investigation of the effects of different NS dosages on the properties of LWAC is highly needed, so as to establish a precise relationship between NS dosage and the fresh and early/long term hardened properties of concrete. Besides, most of the available studies have evaluated the properties of lightweight concretes with densities >1000 kg/m^3^. This study thus proposes and demonstrates several techniques for evaluating the effects of NS on various material properties of LWAC, with a density ranging between 900 and 1000 kg/m^3^. These properties include strength development, thermal conductivity, transport properties (effective water porosity, water absorption coefficient), and evaluation of the air-void system and pore characteristics.

## 2. Materials and Methods

### 2.1. Materials

**Figure 1:**
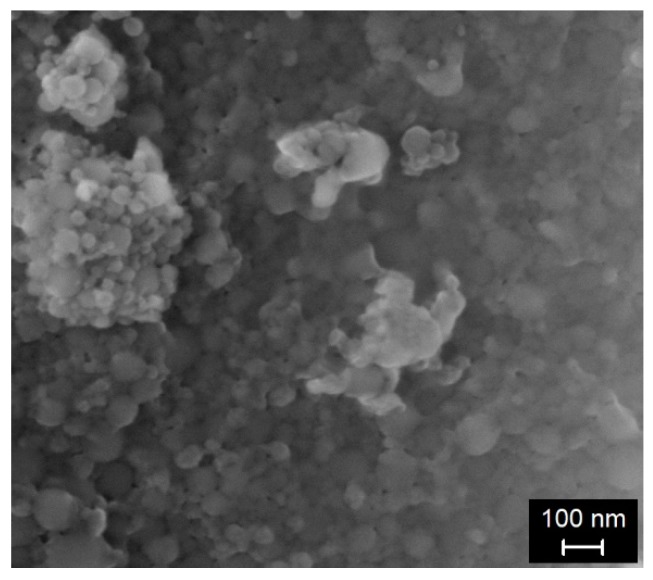
Scanning electron microscope (SEM) micrograph of nanosilica (NS).

This research work studies the influence of nanosilica (NS) addition on the properties of lightweight aggregate concrete (LWAC), with dry density ranges between 900 and 1000 kg/m^3^. To produce the concrete, a commercially available colloidal silica suspension (NS) (Levasil CB8), with a density of 1.4 g/cm^3^, containing 50 wt.% of solid material, was used. The liquid phase in the NS suspension was also considered to be a part of the mixing water, used for the cement paste preparation (see Section 2.2). Scanning electron microscope (SEM, Hitachi S-2700, Tokyo, Japan) micrographs of NS are presented in Figure 1. The SEM images clearly show NS’s spherical shape, with particle sizes <150 nm.

**Figure 2:**
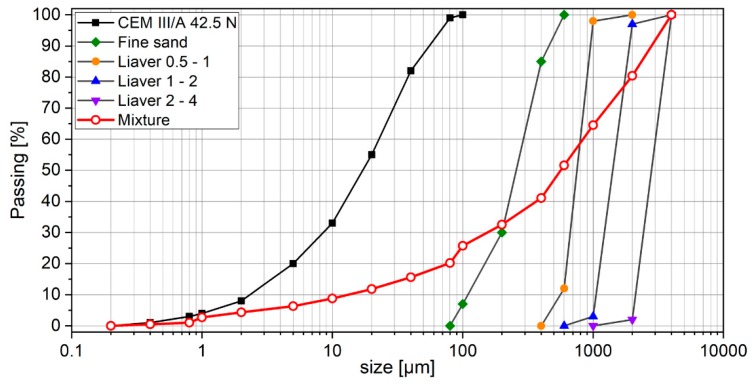
Particle size distributions of concrete constituents and the concrete mixture.

Blast furnace cement, CEM III/A 42.5 N, provided by HeidelbergCement (Heidelberg, Germany), was used in the study. The physical and chemical properties of the cement are summarized in Table 1. Lightweight aggregates—expanded glass (Liaver)—with different fractions (0.5 mm–4.0 mm) were used, with their properties are summarized in Table 2. Because of the low density of expanded glass, it is necessary to increase the amount of fines, to achieve the required dry density. Fine quartz sand (0.1–0.3 mm) was thus added in order to obtain a density between 900 and 1000 kg/m^3^. One of the main problems of lightweight concrete is its sensitivity to compaction and vibration, which can lead to concrete bleeding. A polycarboxylate ether-based superplasticizer (Sika VC 2014), provided by Sika, Germany, was thus used to achieve high workability of the produced concrete; that is, a slump flow >500 mm, according to EN 12350-8. Moreover, owing to the differences in density between the aggregates and the cement paste, segregation is prone to occur in lightweight concrete mixtures and, as such, a viscosity-enhancing admixture (Sika stabilizer ST 10160317) was added to the mixture. The particle size distributions of the concrete constituents as well as measured physical properties of the used expanded glass aggregates are given in Figure 2 and Table 2, respectively. 

### 2.2. Mix Design and Particle Packing Concept

Several parameters significantly affect the characteristics and stability of lightweight aggregate concrete. In this research, the targeted dry density was about 1000 kg/m^3^, and thus lightweight aggregate, rather than normal-weight coarse aggregate, was used. In order to minimize concrete density, it is important to increase the volume of the lightest component in the mixture, which is expanded glass. This can be ensured by applying a packing density concept, without significantly altering the other properties of the lightweight concrete. By applying packing density theory in the mix design, aggregate particles can be packed efficiently in a specific volume while minimizing the gaps between the particles and, consequently, decreasing the amount of paste required to fill voids. The following formula, developed by Andreasen and Andersen [17], was used to calculate the grading of solid particles that gives the maximum packing density of the mixture, as Equation (1):

(1) $$P\left(D\right)=\frac{D^{q}-D_{min}^{q}}{D_{max}^{q}-D_{min}^{q}},$$

where P(D) is the amount of aggregate particle passed through a sieve with size D,$\;D_{max}$ is the maximum aggregate size, $D_{min}$ is the size of the minimum particle, and q is the factor of distribution [18,19]. 

The water absorption of lightweight aggregate is a significant parameter that can affect the properties of concrete, in both fresh and hardened states. To avoid this negative influence, the water absorption of the expanded glass (Liaver) used was determined after 60 min, according to EN 1097-6. The results are presented in Table 2. The aggregates were used in dry conditions, with an additional amount of water equaling the aggregate water absorption added to the mixing water. The w/b ratio for all the mixes was kept constant, at 0.40. The binder content in all the mixes was 500 kg/m³. 

To compare the influence of NS on the properties of LWAC, four mixes were planned and prepared, as presented in Table 3. NS was used as a cement replacement, with ratios of 1 wt.%, 2 wt.%, and 4 wt.%, whereas fine sand was used as a filler for achieving the required density.

A standard concrete mixer with a capacity of 50 liters was used to mix the concrete components as follows. The aggregates, cement, and fine sand were initially dry mixed for 30 s. Next, water, nanosilica, stabilizer, and two-thirds of superplasticizer were added to the mixer, with the mixing process continuing for an additional 3 min. The mixer was then stopped for one minute to control agglomeration and to remove concrete stuck to the bottom of the mixer. Finally, the mixture was mixed for an additional short period of time (30 s), after which the remaining superplasticizer and stabilizer were added, to achieve the target consistency. After mixing, the properties of the fresh concrete were measured for fresh density and consistency. For the hardened concrete, several 10 × 10 × 10 cm^3^ cubical steel molds were filled with concrete to determine its compressive strength, dry density, thermal conductivity, and capillary water porosity. Moreover, 4 × 4 × 16 cm^3^ prisms were also cast to measure the flexural strength and water absorption of the concrete. After casting, the concrete specimens were stored in a laboratory at a controlled humidity and temperature equal to 99% and 20 ± 1 °C, respectively. The samples were demolded after 24 h and cured under water until the day of testing, according to EN 12390-2.

### 2.3. Testing Methods

#### 2.3.1. Oven-Dry Density 

The oven-dry densities of the specimens were measured after 28 days of curing, according to EN 12390-7. Three samples of each case were oven-dried at 105 °C until reaching a constant mass, with the mean value being selected as the dry density. 

#### 2.3.2. Flexural Strength and Compressive Strength

In this investigation, flexural and compressive strength of the produced lightweight concrete were measured at the age of 7, 28, and 90 days to compare the mechanical performance of LWCs with different NS contents. To determine the strengths of the specimens, compression and flexural testing machines (Toni Technik, Berlin, Germany), conforming to EN 12390-3 and EN 196-1, respectively, were used. At the testing day, samples were taken from the curing chamber, and the surface of concrete was dried completely in order to prevent the influence of wetted surfaces on the test results. From each mix, three samples were tested and the mean value was considered.

#### 2.3.3. Thermal Conductivity and Specific Heat

**Figure 3:**
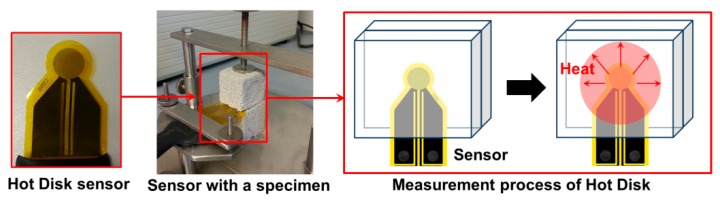
Schematic set-up for measuring thermal conductivity [20].

The thermal conductivity test was performed on 10 × 10 × 10 cm^3^ specimens, using the transient plane source method (Hot Disk, Göteborg, Sweden), conforming to ISO 22007-2 (Figure 3). For each test, two samples were used, and the Hot Disk sensor was laid between the samples. Owing to the high sensitivity of thermal conductivity to the moisture content of concrete, the LWC samples were dried completely at 110 °C until constant mass. After cooling down in moisture-free conditions, the thermal conductivity test was carried out. The test was repeated three times on different samples from each mix, and the mean value was considered. 

#### 2.3.4. Transport Properties

Effective water porosity, in this experiment, is described as the pores that are accessible by water. It was measured simply using the water displacement method. The test was carried out on 10 × 10 × 10 cm^3^ samples. After the curing period, the mass of saturated samples was measured under water (m_sub_). Then, the surfaces of the samples were dried, and the mass of the saturated sample is determined (m_sat_). The samples were dried at 105 °C to constant mass and weighed (m_dry_). From the knowledge of these masses, the effective water porosity can be calculated using the following formula (Equation (2)):

(2) $$Effective\;water\;porosity=\frac{m_{sat}-\;m_{dry}}{m_{sat}-\;m_{sub}}\;\times 100.$$

**Figure 4:**
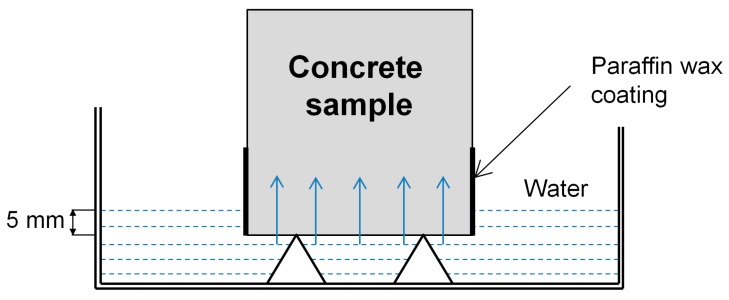
Schematic set-up for measuring the rate of water absorption in concrete specimens.

The water absorption coefficient (water uptake) of the specimens was determined on 4 × 4 × 160 cm^3^ prisms with the partial immersion method, conforming to EN ISO 15148. Prior to the test, specimens were dried to a constant mass, with the sides sealed with hot paraffin wax. During measurement, the water level was kept constant, at about 5 mm above the highest point of the bottom side of the prism (Figure 4). The main force that sucks the water into the concrete sample is the capillary suction. The increase in specimen mass, $\Delta m_{t}$, was determined at specified times up to 24 h and plotted against the square root of the weighing time √t. Afterward, the water absorption coefficient, $W_{w,24h}$, which is described as the ratio between the water quantity absorbed by the specimens per unit area and the square root of time, was calculated.

#### 2.3.5. Air-Void Analysis

**Figure 5:**
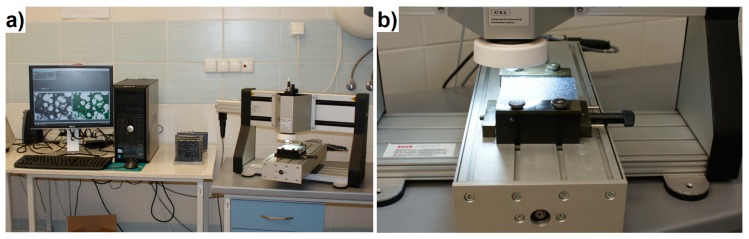
RapidAir 457 device (**a**), specimen during measurement (**b**).

To evaluate the air-void characteristics of the concrete specimens, automated image analysis systems based on a linear traverse method (conforming EN 480-11) were performed, with the use of a RapidAir 457 Automated-Air-Void-Analyzer produced by Germann Instruments A/S, Copenhagen Denmark (Figure 5a). This method can be successfully applied to analyze the air-void characteristics of both normal- and lightweight concretes [21,22,23,24]. Air-voids measured from RapidAir have the size larger than 10 µm, which is the maximum available resolution of the device. Cured concrete samples were cut with a diamond saw into 1 cm thick slices having a size of 10 × 10 cm² at the middle of the specimen. These were then polished, using different grit sizes (from 600 to 1500), so as to ensure a smooth specimen surface before preparation. In the next step, the surfaces of the samples were firstly painted with a broad nib black marker and then heated to 55 °C, after which white zinc paste was applied on the surface of the specimens. After cooling, the residual powder was removed from the surface of the specimens. Two specimens were tested for each case, with each sample being tested twice (Figure 5b). 

#### 2.3.6. Microstructural Pore Analysis

Pore structure characteristics were determined with the use of the mercury intrusion porosimetry (MIP) method. MIP measurement made it possible to focus on pore sizes <10 µm. In general, it is known that MIP has disadvantages when measuring relatively large voids [20]; it is thus used for small pores, whereas larger voids can be measured more effectively using RapidAir. An MIP test was performed on small-cored samples, taken from the core of the concrete’s cube, in order to obtain information regarding the pore size distributions of the concrete specimens. The specimens were immersed in isopropanol after 28 days of curing to stop hydration, after which they were freeze-dried prior to testing. Furthermore, scanning electron microscopy (SEM) was also applied, to evaluate the microstructural characteristics of the concrete, with small samples from the core of the concrete cube cut out, using a diamond blade, after 28 days of curing. The samples were then polished using sandpaper. 

## 3. Results

### 3.1. Fresh and Oven-Dry Density

LWAC is highly sensitive to any changes in its constituents. Increasing the water or superplasticizer dosages can lead to segregation and bleeding. In the experimental part of this research, superplasticizer dosages were adopted and adjusted to achieve the required consistency class, without negatively affecting concrete homogeneity and stability. As can be seen from Table 3, superplasticizer dose increased with NS content. Simultaneously decreasing stabilizer dosages with increments in NS content was required to achieve reasonable flowability. The incorporation of NS into mixtures does not noticeably affect fresh and oven-dried specimen densities (Table 3), with all values obtained being within the experimental error.

### 3.2. Thermal Conductivity and Specific Heat

**Figure 6:**
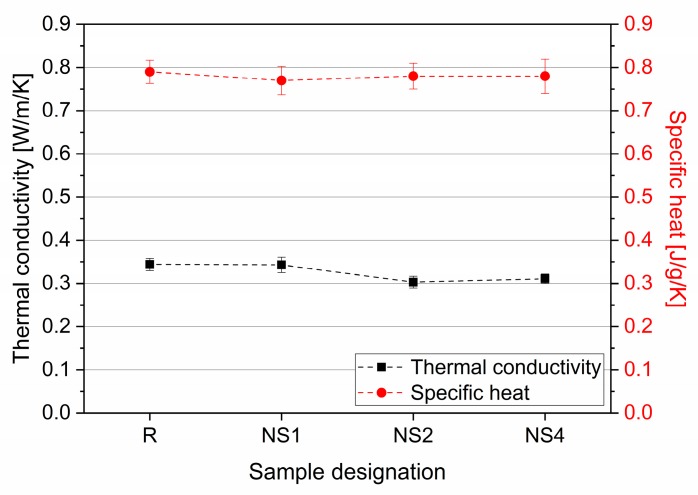
Thermal conductivity and specific heat of tested specimens.

Figure 6 represents the results of thermal conductivity and specific heat tests of the concrete specimens. No significant differences between either thermal conductivity or specific heat were observed between the specimens, with only a minimal decrement in the thermal conductivity of specimens containing a higher amount of NS being reported. All specimens tested exhibited relatively low thermal conductivity values (lower than 0.35 W/m/K).

### 3.3. Flexural Strength and Compressive Strength

**Figure 7:**
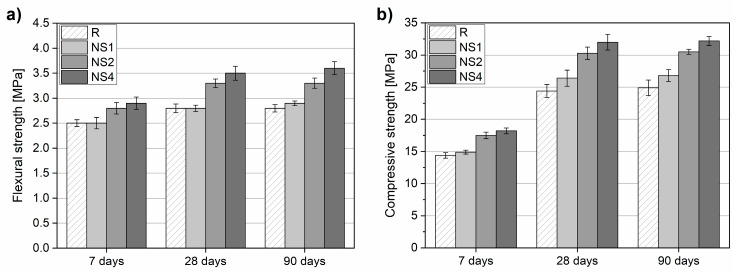
Flexural strength (**a**) and compressive strength (**b**) after 7, 28, and 90 days of curing.

Flexural strength and compressive strength results are depicted in Figure 7. A beneficial effect on flexural strength development was observed in the case of 2 wt.% and 4 wt.% NS addition (Figure 7a). Specimens NS2 and NS4 exhibited higher flexural strength values after 7 and 28 days of curing, by 12%, 16%, 18%, and 25%, respectively, in comparison with the reference mix. In the case of low NS dosage (specimen NS1), no significant effect on flexural strength was observed. After 90 days of curing, the specimens exhibited strength comparable to that of 28 day flexural strength.

In the case of compressive strength, the incorporation of NS at all dosages had a beneficial effect on all concretes tested (Figure 7b). Strength increased gradually with the amount of NS added to the mixture. After 7 days of curing, NS1, NS2, and NS4 exhibited 4%, 22%, and 26% higher compressive strengths, respectively, than specimen R. After 28 days of curing, the strength ratio between pristine and NS-modified specimens increased. In the case of a lower NS dosage (NS1), strength improvement was relatively limited (improvement by 8%), while NS2 and NS4 exhibited strength improvements of 24% and 31%, respectively. After 90 days of curing, similarly to flexural strength, specimens exhibited only marginal compressive strength improvements in reference to 28 day strength. 

### 3.4. Effective Water Porosity and Water Absorption Coefficient

**Figure 8:**
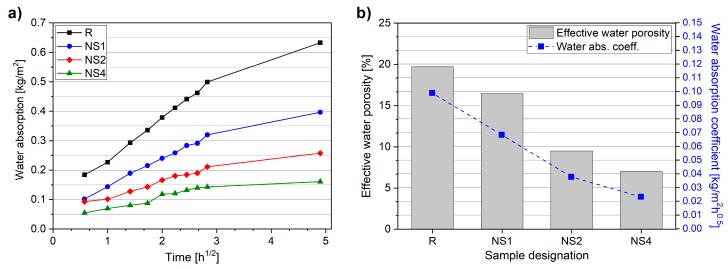
Capillary absorption curve (**a**), effective water porosity and water absorption coefficient (**b**).

Figure 8 summarizes the results of effective water porosity and water absorption coefficient measurements. It can be clearly seen in Figure 8a that the water absorption rates were significantly different and highly dependent on the amount of NS used. The water absorption coefficient decreased noticeably with an increment in the amount of NS, with the NS4 specimen exhibiting a water absorption coefficient value over four times lower than the R specimen. Similarly, the effective water porosity value decreased with increasing NS dosage, and a substantial decrement of effective water porosity was observed when >1 wt.% of NS was incorporated. The effective water porosity was decreased from 19.7% for specimen R to 16.5%, 9.5%, and 7.0% for specimens NS1, NS2, and NS4, respectively.

### 3.5. Air-Voids Characteristics

**Figure 9:**
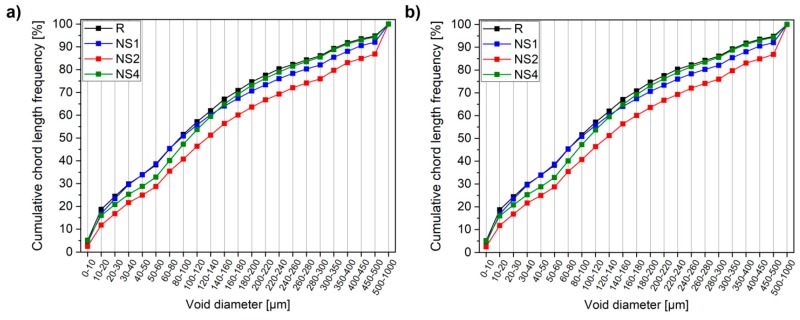
Total porosity (**a**) and cumulative chord length frequency (**b**) of concretes determined with a RapidAir device.

The relationship between the amount of NS used and the air-void characteristics was determined on the basis of a 2D image analyzing technique, with the use of a RapidAir 457 testing device. The air-void characteristics are presented in Figure 9, with the selected parameters summarized in Table 4.

It can be seen that the total porosities (Figure 9a) of the concretes tested are relatively similar, with only specimen NS1 exhibiting a slightly lower total porosity value in comparison with pristine concrete (R). Despite the comparable total porosity values, it is clear that the total number of voids distinguished by the RapidAir system in NS-incorporated specimens was significantly lower than in pristine concrete (Table 4). This implies that the amount of larger diameter voids increased, which in turn resulted in comparable total porosities. It is widely known that voids of a higher diameter contribute more significantly to total porosity. It is important to note that this trend was observable until the addition of 2 wt.% of NS, while the incorporation of 4 wt.% of NS resulted in an increment in the number of voids distinguished. This phenomenon was also confirmed by the cumulative chord length frequency (Figure 9b), where all specimens containing NS exhibited a lower amount of chords with a small diameter. On the basis of the results presented in Table 4, it can be assumed that a decrement in the number of voids in NS-incorporated specimens denotes a high possibility of the existence of more solid content between voids. Overall, this implies that NS-incorporated specimens possess a more refined or robust void structure than pristine concrete (R).

### 3.6. Mercury Intrusion Porosimetry (MIP)

**Figure 10:**
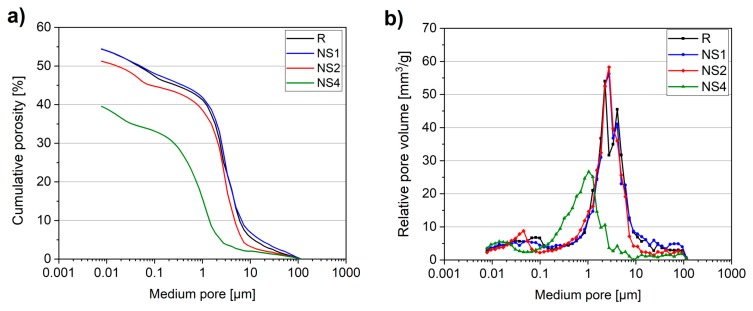
Cumulative porosity (**a**) and relative pore volume (**b**) of concretes obtained with the mercury intrusion porosimetry (MIP) test.

As the RapidAir device is well suited to evaluating the air-void characteristics of concrete, the mercury intrusion porosimetry (MIP) method can be successfully applied to evaluating the pore size distribution and the fine pore structure of cement matrices, thus providing insight into their microstructural characteristics, which are responsible for the durability and transport-related properties of concrete. Figure 10 and Table 5 present the results of pore structure characteristic tests. The MIP results confirmed the high porosity of the lightweight concrete matrix (Figure 10a). In addition, it can be seen that the incorporation of NS contributed to a substantial reduction in concrete microstructure pore diameters. No noticeable effect was observed in the case of low nanosilica dosages (NS1), with the specimen exhibiting almost the same porosity as the control specimen. However, a further increment in NS dosage contributed to noticeable changes in concrete microstructure. The total porosity decreased from 54.38 vol.% (R) to 51.23 vol.% and 39.53 vol.% for specimens NS2 and NS4, respectively. It can also be seen that the pore size distribution of NS modified specimens shifted to smaller pores (Figure 10b); thus, the amount of coarser pores decreased, which relates to a decrement in the average and median pore diameters of NS-modified specimens.

### 3.7. Scanning Electron Microscope (SEM) Analysis

**Figure 11:**
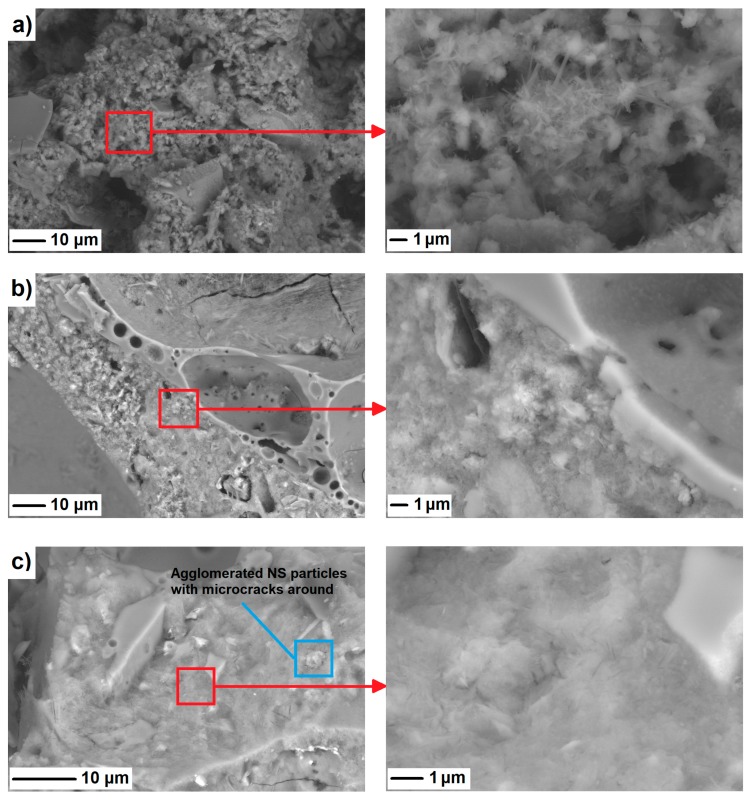
Scanning electron microscope micrographs of R (**a**), NS2 (**b**), and NS4 (**c**) after 28 days of curing.

For a further understanding of the role of NS in the refinement of LWAC’s microstructure, a SEM analysis was also undertaken. The measurements were performed on concrete samples at the age of 28 days. Figure 11 presents the SEM images of the reference mix (R) and NS-incorporated specimens (NS2 and NS4). It is clear from the images that the incorporation of NS densified the microstructure. This can be attributed to both the filling effect and the pozzolanic reactivity of NS particles. The latter resulted in a significant reduction in the number of voids, as reflected in the results of the RapidAir measurements. However, the pozzolanic reaction of nanoparticles with portlandite created additional calcium silicate hydrates (C–S–H), which replaced the portlandite sheets and refined the microstructure. Moreover, with the addition of an excessive amount of nanosilica to the mixture (specimen NS4), agglomeration of NS particles can be observed in the SEM images (Figure 11c). At higher NS dosages, very fine particles tended to agglomerate, with microcracking occuring afterwards around the agglomerated particles, owing to volumetric changes associated with drying. As a result of such agglomeration, so-called weak-zones are produced in concrete, suppressing further increments in its mechanical performance (which was also reported in this study). 

## 4. Discussion

On the basis of the results presented above, it can be concluded that NS significantly modifies the plastic and hardened properties of LWACs. A beneficial effect on mechanical and transport properties was clearly observed. However, the incorporation of NS requires certain mixture modifications, to produce concrete with satisfactory plastic properties, so as to obtain the appropriate hardened properties. Generally speaking, the effect of NS on the fresh properties of normal-weight concrete has been studied widely by several researchers [25,26,27]. It has been reported that NS affects the workability of concrete by decreasing consistency and by increasing the viscosity of fresh mixtures. LWAC is highly sensitive to changes to its constitution, and thus increasing water or superplasticizer dosages can lead to segregation and bleeding [25]. As shown above (Table 3), the SP dose increases with the NS content. This is because of the high fineness of NS particles and the tendency of fine particles to agglomerate. As a result of the very high surface area to volume ratio of NS, particles exhibit a higher water demand, meaning that less mixing water is available for the other concrete constituents and that higher SP dosages are thus required in the mix. However, a simultaneous decrement in the amount of stabilizer required was observed. This phenomenon can be attributed to the effects of NS, which increases mixture viscosity, thus improving particle cohesiveness and preventing concrete segregation. The simultaneous optimization of superplasticizer and stabilizer dosages, therefore, results in a stable self-compacting mixture. From these observations, it can be concluded that NS has a remarkable effect on increasing mixture cohesiveness, and thus preventing segregation. A similar phenomenon was observed in the work of Afzali Naniz and Mazloom [28], where NS-incorporated self-compacting LWAC (with a density of <1800 kg/m^3^) initially required a higher amount of superplasticizer than pristine concrete, but in turn, no bleeding in NS-modified mixtures was observed. 

Changes in mixture design, through the incorporation of NS, did not contribute to a noticeable alteration of either fresh or dry specimen densities. Similarly, no noticeable differences between thermal conductivity or specific heat values were observed. As has been widely reported, thermal conductivity is highly related to LWAC density, with mixture composition having only a minor effect on this parameter [29,30,31]. However, some authors [32,33,34] have reported that NS and SF might contribute to a decrement in the thermal conductivity of cement-based composites (concretes, mortars, and pastes), but this effect increases only when a higher amount of admixture/additive is incorporated. Nevertheless, in this work, we did not observe any significant changes to these values. 

In the case of the hardened properties of LWAC, it was observed that even small NS dosages (1 wt.%) were beneficial in improving overall concrete performance, with increments in NS content leading to considerable improvements. The beneficial effect of NS on strength development can be attributed to the nano-filling and nucleating effects, as well as to the pozzolanic activity of silica nanoparticles, which results in an acceleration of cement hydration, the production of a higher amount of dense calcium–silicate–hydrate (C-S-H) phase, a refinement in microstructure, and an improvement in the interfacial transition zone (ITZ) between the cement matrix and the aggregate [35,36,37,38,39]. As a result, the strength development process is accelerated from the early hours of hydration, together with an improvement in long-term mechanical properties [40,41,42,43]. The beneficial influence of NS addition on both the flexural and compressive strengths of LWAC was clearly visible in this study from early stages (7 day), and the accelerating effect of NS on cement hydration was confirmed. At an age of 28 days, a further improvement in flexural and compressive strength, proportional to the NS dose, was observed, while after 90 days, the strength values remained comparable to that of 28 day strength. As such, the addition of NS significantly increased the strength of the transition zone, improving the bond between the cement matrix and the aggregates, while densifying the LWAC transition zone. 

The improved mechanical performance of LWAC can also be attributed to an alteration in void characteristics, when NS was present in the mixture. The incorporation of NS contributed to decreasing the total number of voids in the mixes and to an increase in the production of voids with a slightly larger diameter. This resulted in maintaining relatively similar total porosity, along with increased average chord length and spacing factor, thus resulting in the production of robust structures with increased mechanical performance. 

In addition, incorporation of NS significantly contributed to the refinement of the pore structure in the capillary range. Previous studies [14,44,45] have reported that NS particles strongly affect the reduction of the size of capillary pores. Simultaneously, the use of NS tends to increase the proportion of smaller pores, by disconnecting continuous pores from discontinuous ones and subdividing larger pores into smaller ones; thus producing a material with a finer pore structure, which exhibits lower transport properties. The MIP measurements in the present study, as well as those of other authors [36,46,47,48], confirm that total porosity decreases significantly in the presence of NS in the cement matrix, when the optimal amount of NS is incorporated. Therefore, as reported in this study, NS-modified composites exhibit denser microstructures with lower water absorption, which might potentially prevent the ingress of aggressive solutions to the pore structure [12,14,49,50,51]. However, it should be highlighted that exceeding the optimal amount of NS can contribute to worsening some concrete properties; that is, mechanical properties (via the creation of agglomerates), despite the positive effects of the presence of NS on other properties (transport properties). Because of its very small particle size, nanosilica tends to agglomerate, and homogeneous distribution of particles is difficult to achieve. Therefore, an optimum dosage of nanosilica, above its negative influences on concrete characteristics, should be carefully considered. The results obtained in this investigation prove that the mechanical properties and impermeability of concrete are enhanced up to 4 wt.% addition of nanosilica to the lightweight concrete mixture. Above the optimal limit, the nanosilica particles clump and can lead to a significant reduction in mechanical properties. 

On the basis of the results obtained in this work, it is clear that the incorporation of NS has a beneficial effect on the mechanical and transport properties of LWAC. However, to utilize NS beneficially in the production of LWACs, a proper mix design—with the optimal amounts of NS, SP, and ST meeting both economic and mechanical/durability requirements—is required. 

## 5. Conclusions

From the results obtained, the following conclusions can be drawn:

- Superplasticizer dosage required to achieve the targeted consistency increases with the nanosilica content owing to the large surface area and small nanosilica particles.
- NS has a beneficial effect on the early flexural and compressive strengths of LWACs, with the effect increasing with NS dosage. When a lower amount of NS is incorporated (1 wt.%), strength improvement is relatively limited.
- NS added at a dosage of >1 wt.% effectively increases the 28 day flexural and compressive strengths. After 90 days, further strength improvements were not observed.
- The remarkable decrement of effective water porosity and the water absorption coefficient was analyzed in specimens containing NS. This effect increased with an increment in NS content, and best performance was exhibited by specimen containing 4 wt.%.
- The incorporation of NS leads to compaction and refinement of air-void structures in LWAC; thus more robust lightweight concrete structures are obtained. In addition, the presence of NS in the mixture refines the fine pore structure, resulting in an improvement in transport properties.

## Figures and Tables

**Table 1:** Chemical and physical characteristics of cement.

Material	CaO	SiO_2_	Al_2_O_3_	Fe_2_O	MgO	Na_2_O	K_2_O	SO_3_	Specific Density (g/cm^3^)	Surface Area (Blaine) (cm^2^/g)
	(wt.%)									
CEM III/A 42.5N	54.8	23.7	8.8	1.46	5.21	0.3	0.06	2.4	3.05	3860

**Table 2:** Measured physical properties of the lightweight aggregate (LWA).

Material	Bulk Density (kg/m^3^)	Specific Density (kg/m^3^)	Crushing Resistance (MPa)	Water Absorption 60 min (wt.%)
LWA 0.5–1.0	250	450	≥2.6	12.08
LWA 1.0–2.0	220	330	≥2.4	13.96
LWA 2.0–4.0	190	310	≥2.2	11.50

**Table 3:** Composition of lightweight concrete mixes.

Mix	Mix Proportions [kg/m^3^]									Fresh Density [kg/m^3^]	Oven-Dry Density [kg/m^3^]
	Cement	NS ^1^	Water	Quartz Sand 0.1–0.3 mm	Liaver 0.5–1 mm	Liaver 1–2 mm	Liaver 2–4 mm	SP ^2^	ST ^3^		
**R**	**500**	-	200	280	71.8	49.22	51	3.1	1.32	1097	945
NS1	495	10	195	280	71.8	49.22	51	4.8	0.94	1089	950
NS2	490	20	190	280	71.8	49.22	51	5.8	0.22	1063	915
NS4	480	40	180	280	71.8	49.22	51	9.6	0.22	1108	955

^1^ NS: nanosilica suspension (50 wt.% of solid mass); ^2^ SP: superplasticizer; ^3^ ST: stabilizer.

**Table 4:** Characteristics of air-voids in specimens.

Mix	Number of Voids	Total Porosity [%]	Average Chord Length [mm]	Spacing Factor [mm]	Specific Surface [mm^−1^]	Void Frequency [mm^−1^]
R	6617	32.9	0.145	0.027	27.63	2.742
NS1	5524	28.3	0.161	0.033	24.89	2.289
NS2	5587	31.9	0.193	0.032	20.69	2.315
NS4	5958	31.7	0.154	0.030	25.91	2.469

**Table 5:** Results of the mercury intrusion porosimetry (MIP) measurements.

Mix	R	NS1	NS2	NS4
Total porosity [vol.%]	54.38	54.40	51.23	39.53
Median pore diameter [μm]	2.498	2.321	2.277	0.686
